# Efficacy of the *‘Stand and Move at Work’* multicomponent workplace intervention to reduce sedentary time and improve cardiometabolic risk: a group randomized clinical trial

**DOI:** 10.1186/s12966-020-01033-3

**Published:** 2020-10-27

**Authors:** Mark A. Pereira, Sarah L. Mullane, Meynard John Lapore Toledo, Miranda L. Larouche, Sarah A. Rydell, Brenna Vuong, Linda H. Feltes, Nathan R. Mitchell, Junia N. de Brito, Kristina Hasanaj, Neil G. Carlson, Glenn A. Gaesser, Noe C. Crespo, J. Michael Oakes, Matthew P. Buman

**Affiliations:** 1 University of Minnesota, School of Public Health, 1300 South Second Street, Minneapolis, MN 55455 USA; 2 Arizona State University, College of Health Solutions, 500 North 3rd Street, Phoenix, AZ 85004 USA; 3 Fairview Health Services, Minneapolis, MN USA; 4 Minnesota Department of Health, Minneapolis, MN USA; 5 San Diego State University, San Diego, CA USA

## Abstract

**Background:**

Sedentary time is associated with chronic disease and premature mortality. We tested a multilevel workplace intervention with and without sit-stand workstations to reduce sedentary time and lower cardiometabolic risk.

**Methods:**

*Stand and Move at Work* was a group (cluster) randomized trial conducted between January 2016 and December 2017 among full-time employees; ≥18 years; and in academic, industry/healthcare, and government worksites in Phoenix, Arizona and Minneapolis/St. Paul, Minnesota, USA. Eligible worksites were randomized to (a) *MOVE+*, a multilevel intervention targeting reduction in sedentary time and increases in light physical activity (LPA); or (b) *STAND+*, the *MOVE+* intervention along with sit-stand workstations to allow employees to sit or stand while working. The primary endpoints were objectively-measured workplace sitting and LPA at 12 months. The secondary endpoint was a clustered cardiometabolic risk score (blood pressure, glucose, insulin, triglycerides, and HDL-cholesterol) at 12 months.

**Results:**

Worksites (*N* = 24; academic [*n* = 8], industry/healthcare [*n* = 8], and government [*n* = 8] sectors) and employees (*N* = 630; 27 ± 8 per worksite; 45 ± 11 years of age, 74% female) were enrolled. All worksites were retained and 487 participants completed the intervention and provided data for the primary endpoint. The adjusted between arm difference in sitting at 12 months was − 59.2 (CI: − 74.6,-43.8) min per 8 h workday, favoring *STAND+*, and in LPA at 12 months was + 2.2 (− 0.9,5.4) min per 8 h workday. Change in the clustered metabolic risk score was small and not statistically significant, but favored *STAND+.* In an exploratory subgroup of 95 participants with prediabetes or diabetes, the effect sizes were larger and clinically meaningful, all favoring *STAND+,* including blood glucose, triglycerides, systolic blood pressure, glycated hemoglobin, LDL-cholesterol, body weight, and body fat.

**Conclusions:**

Multilevel workplace interventions that include the use of sit-stand workstations are effective for large reductions in sitting time over 12 months. Among those with prediabetes or diabetes, clinical improvements in cardiometabolic risk factors and body weight may be realized.

**Trial registration:**

ClinicalTrials.gov Identifier: NCT02566317. Registered 2 October 2015, first participant enrolled 11 January 2016.

Replacing sedentary time with standing and light-intensity physical activity (LPA) is associated with improved cardiometabolic health and lower mortality risk, [1, 2] especially among those not sufficiently engaged in moderate-vigorous physical activity (MVPA) [3]. As a consequence of increased automation and reliance on computers, [4] workers in the U.S. and most developed countries spend 70–80% of their work time sedentary [5, 6]. Interventions targeting reductions in workplace sedentary time have yielded cautionary, yet promising findings [7] and the U.S. Physical Activity Guidelines Advisory Committee has called for more rigorous clinical trials [8]. Interventions that are multicomponent in nature and include individual-level strategies (e.g., education, motivational support) with environmental changes (i.e., sit-stand workstations) have produced the largest reductions in sedentary time [9]. However, the overall quality of these studies is limited by nonrandomized designs, short follow-up (< 6 months), and small samples. Two recent group randomized trials have addressed some of these weaknesses, [6, 10] employing randomization at the workgroup level and following for 12 months, and demonstrating large reductions in sedentary time (~ 45 min/8 h workday). However, they were limited by (a) low power to detect changes in health outcomes; (b) lack of diversity among worksites, limiting generalizability; and (c) lack of comparison to an active intervention, a necessary element to support policy decisions [11].

We aimed to address these limitations by testing the *Stand and Move at Work* intervention*,* a 12-month multilevel workplace intervention that included the use of sit-stand workstations and workplace policy, environmental, social, and individual-level changes. We compared this intervention to a similar multilevel intervention that did not include sit-stand workstations. Our primary outcome was objectively-measured workplace sitting and LPA. Our secondary outcome was a clustered cardiometabolic risk score. We hypothesized the inclusion of sit-stand workstations to a multilevel intervention would result in less workplace sitting, more LPA, and more favorable changes in cardiometabolic risk relative to the comparison condition.

## Methods

### Participants

Clinical trial registration number is NCT02566317. Worksite eligibility criteria were: (a) small to moderate workgroup size (i.e., 20–60 employees); (b) > 80% of employees working full time; (c) predominantly seated desk-based office work; (d) not currently undergoing a worksite wellness program to reduce sitting or increase LPA; (e) < 10% of employees using a sit-stand workstation; (f) willing to have sit-stand workstations installed; and (g) leadership willing to be randomized to either study arm. Worksites were recruited in the Phoenix, AZ and Minneapolis/St. Paul, MN, USA greater metropolitan regions and were selected using purposive sampling across academic, industry/healthcare, and government sectors. Employee eligibility were: (a) 18 years or older; (b) generally good health and able to safely reduce sitting and increase LPA; (c) working full-time on-site; (d) not currently pregnant; (e) predominant worksite occupation requiring seated office work; (f) not currently using a sit-stand workstation; (g) willing to have a sit-stand workstation installed at their desk; and (h) willing to be randomized to either study arm. Employees completed screening via questionnaire followed by in-person adjudication. Full details of recruitment strategies are published [12]. Worksites were identified through contacts with worksite wellness professionals in the regions. Employees were recruited through town halls hosted by the employer. The Arizona State University and the University of Minnesota Institutional Review Boards approved the study protocol, and individuals signed informed consent prior to baseline.

### Study design

This study was a two-arm group-randomized trial. Worksites were randomized to one of two interventions: (a) *MOVE+*, a multilevel behavioral intervention targeting increases in LPA at the worksite; or (b) *STAND+*, the *MOVE+* intervention along with newly installed sit-stand workstations to allow employees to sit or stand at their desks while working. A simple randomization procedure was used following stratification among the three sectors (i.e., academic, industry/healthcare, and government) nested within each of the two regions (i.e., Phoenix, AZ and Minneapolis/St. Paul, MN), which was performed by the study biostatistician. Four worksites were enrolled every 2 months between January 2016 and November 2016 to avoid seasonal effects. Twelve-month outcome data were collected between February 2017 and December 2017.

### Interventions

The “*Stand and Move at Work*” interventions were multi-component interventions designed to reduce sitting and increase LPA at work. Both interventions were actively delivered for 12 months, targeted the workplace, and were drawn from the social ecological model [13]. Levels targeted were the individual (education, behavioral cues, goal setting), social environment (group cooperation, contests, role modeling), physical environment (signage, centrally located printers and waste bins), and workplace policies (managerial support, new policies, worksite sponsored messaging). The full descriptions of these multi-component interventions are published [14]. The *MOVE+* intervention had a primary goal of ≥30 min of additional LPA throughout the workday, an achievable goal with modest health benefit [2, 3]. The *STAND+* intervention had the same LPA target as *MOVE+*, but with an additional goal of increasing standing time to 50% of desk-based worktime. All participating employees in the *STAND+* arm had an Ergotron Workfit-TL model sit-stand workstation (Ergotron, Inc., St. Paul, MN) installed at their work desk. Workstations were installed by trained research staff and ergonomic consultation was provided. *MOVE+* worksites had workstations installed following the 12-month active intervention period.

### Measures

All assessments were completed at 0 (baseline), 3 (interim), and 12 (posttest) months. Clinical and biomarker assessments were conducted at each worksite. Questionnaires were administered online (Qualtrics, Salt Lake City, UT).

#### Demographic variables

Age, race, sex, education, and job type were assessed.

#### Workplace sitting time

The activPAL3c micro accelerometer (PAL Technologies Limited, Glasgow, United Kingdom) was used to assess sitting and physical activity during work and nonwork times over seven consecutive days. The activPAL is a small, thigh-worn sensor that is valid for distinguishing sitting from standing positions and for classifying time spent in physical activity [15, 16]. The activPAL was waterproofed using medical grade adhesive and attached to the midline of the thigh using a breathable, hypoallergenic tape. Participants were instructed to wear the device 24 h/day without removing for bathing or other water-based activities. Exceedingly long bouts of continuous sedentary or standing time (> 6 h) were considered non-wear time and excluded from analyses. Sleep periods were excluded using time in bed reports from a daily log when available, and an automated algorithm when not available [17]. Wake periods with ≤10 h of wear time were excluded. Work periods with < 4 h of wear time were also excluded. Outcomes are reported as work periods (as the intervention targeted this period only) and total wake time (to assess possible compensation effects outside of work). Work periods were standardized to an 8 h workday (i.e., standardized minutes = observed minutes × 480/observed minutes of wear time). Waking periods were standardized to a 16 h day. The following outcomes are reported: sitting (min/day); standing (min/day); LPA (min/day); MVPA (min/day); total physical activity, combined LPA and MVPA (min/day); sit-to-stand transitions (number of transitions/h of sitting); and sitting time accrued in bouts ≥30 m (min/day).

#### Cardiometabolic risk biomarkers

Body weight, resting blood pressure, and fasting and venous serum concentrations of glucose, insulin, triglycerides, and LDL- and HDL-cholesterol were assessed using standard procedures previously described [14]. All biomarker samples were batch-processed in triplicates in the Advanced Research and Diagnostic Laboratory at the University of Minnesota. To assess cardiometabolic risk in a continuous fashion, a summary metabolic risk score (CMR) was calculated by summing z-scores for each component of the metabolic syndrome [18]. The HDL z-score was subtracted rather than added and diastolic and systolic blood pressure z-scores were averaged prior to addition. The CMR was the secondary outcome; however, both the CMR score and individual biomarker results are presented. In an exploratory analysis, a subgroup of “dysglycemic” high-risk participants were identified based on either a previous diabetes diagnosis or a fasting blood glucose ≥100 mg/dL. A hemoglobin A1c assay was completed in this subgroup. As this subgroup included diagnosed and undiagnosed diabetes, we did not analyze insulin in this subgroup, nor did we include it in the CMR. This subgroup analysis was chosen given the stronger effects in the epidemiological literature on the role of sitting time in glucose regulation outcomes compared to other outcomes [19].

### Sample size and statistical analysis

**Fig. 1:**
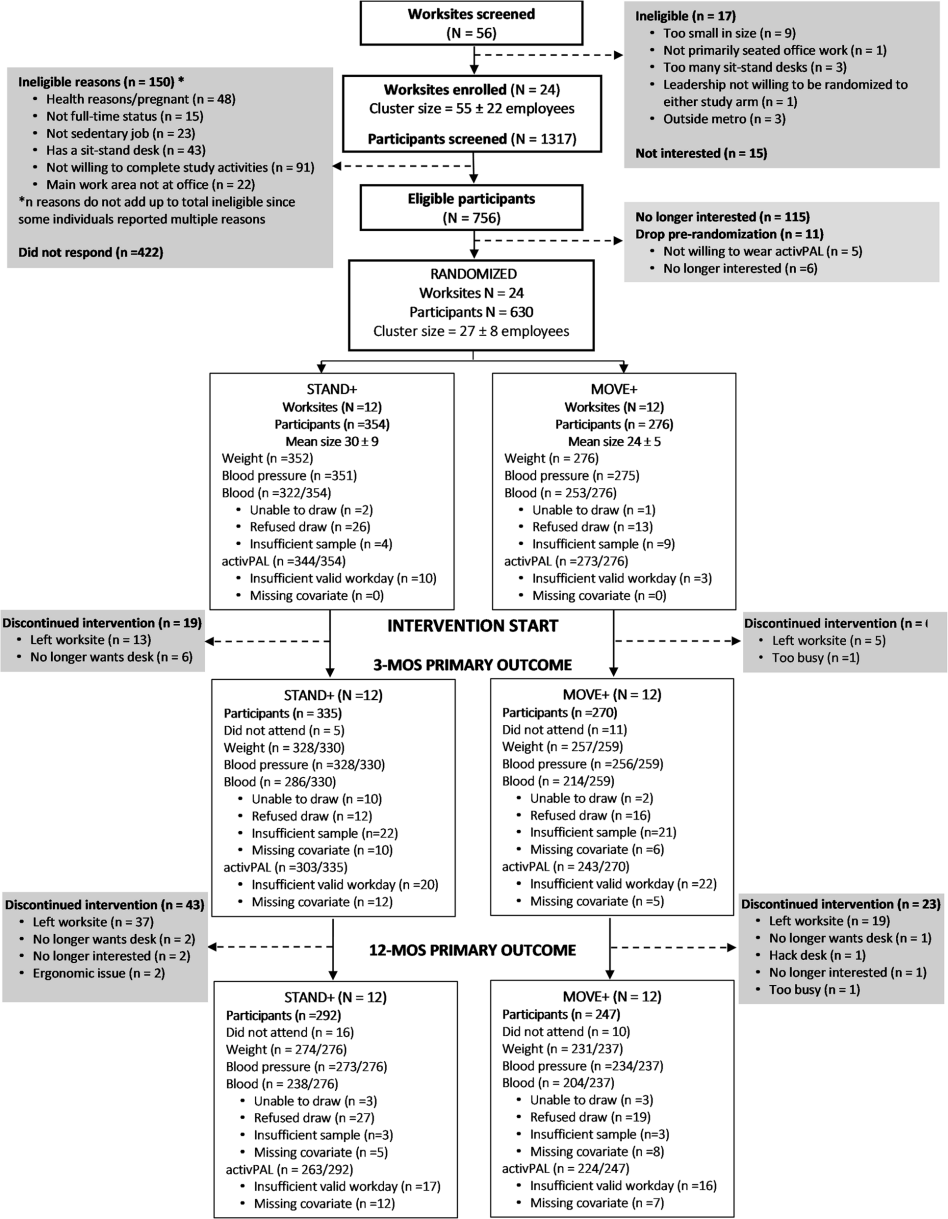
Worksite and participant flow

Analyses were performed in SAS 9.4 (SAS Institute Cary, N.C.). Intent to treat procedures were followed at the level of the worksite (the unit of randomization), with 24 worksites being randomized and analyzed. Individuals within worksite were included when baseline and follow-up outcome data were available. The CONSORT in Fig. 1 describes the reasons for loss-to-follow-up at the individual level. The senior statistician was blinded to group assignment and the analyst/programmer was blinded until the statistical models were finalized. Individuals who became pregnant or lactating during the trial were excluded from cardiometabolic analyses. Each outcome was defined as change from baseline. Distributions were examined and plotted against baseline to identify implausible values and influential points prior to analysis. Sensitivity analyses using log transformed and winsorized (3rd quartile + 1.5*SD) outcomes gave similar results and are not reported. Linear mixed models evaluated within group changes and between group differences. Effects were tested separately using 3- and 12-month outcomes. The group randomized design was accounted for using a random effect for site nested within treatment. Models were adjusted for baseline values of the respective outcome and a priori selected covariates: age, sex, race/ethnicity, and baseline BMI. The study was designed to have 80% power (alpha = 0.05) to detect a difference of 14.6 min per 8 h workday of sitting and 12.2 min per 8 h workday of LPA between intervention arms.

## Results

Twenty-four worksites (12 per study arm; N workers = 630) were included in the baseline examination. Four worksites were recruited and retained in each region/sector stratum. Figure 1 presents the CONSORT diagram for recruitment, randomization, and retention. All worksites were retained and 487 participants completed the 12-month intervention and provided adequate data for the primary outcome analysis, an overall retention rate of 77% (72% in Phoenix and 83% in Minneapolis/St. Paul). The retained worksite sample size was 18.7 ± 5.3 participants (min = 12, max = 27) for *MOVE+*, and 21.9 ± 7.2 (min = 15, max = 39) for *STAND+*. Table 1 describes the baseline characteristics of the randomized participants by study arm. Supplementary Table 1 describes baseline characteristics for the analyzed sample only. Supplementary Table 2 describes baseline cardiometabolic biomarkers for the dysglycemic subgroup.

**Table 1:** Baseline demographics, behavioral out

	*Stand+*	*Move+*	Total
	n (%)	n (%)	n (%)
N worksites	12 (50.0)	12 (50.0)	24 (100.0)
N individuals	354 (56.2)	276 (43.8)	630 (100.0)
Region			
Phoenix, Arizona	194 (54.8)	138 (50.0)	332 (52.7)
Minneapolis/St. Paul, Minnesota	160 (45.2)	138 (50.0)	298 (47.3)
Age (years)	45.6 ± 11.4	43.3 ± 10.8	44.6 ± 11.2
Race			
Non-Hispanic White	239 (67.5)	205 (74.3)	444 (70.5)
Hispanic	57 (16.1)	30 (10.9)	87 (13.8)
Non-Hispanic Black	20 (5.7)	6 (2.2)	26 (4.1)
Non-Hispanic Asian	15 (4.2)	17 (6.2)	32 (5.1)
Other/Multiracial/Unknown	23 (6.5)	18 (6.5)	41 (6.5)
Female	296 (83.6)	173 (62.7)	469 (74.4)
Education			
Less than college	14 (4.0)	15 (5.4)	29 (4.6)
College/Some college	234 (66.1)	151 (54.7)	385 (61.1)
Graduate/Professional	93 (26.3)	98 (35.5)	191 (30.3)
Unknown	13 (3.7)	12 (4.4)	25 (4.0)
Work sector			
Academic	119 (33.6)	94 (34.1)	213 (33.8)
Industry/healthcare	123 (34.8)	83 (30.1)	206 (32.7)
Government	112 (31.6)	99 (35.9)	211 (33.5)
Job type			
Executive	43 (12.2)	39 (14.1)	82 (13.0)
Professional	182 (51.4)	155 (56.2)	337 (53.5)
Clerical	120 (33.9)	72 (26.1)	192 (30.5)
Behavioral Outcomes			
Work periods (min per 8 h workday)			
Sitting	330.8 ± 79.8	337.9 ± 73.1	334.0 ± 76.9
Standing	113.6 ± 75.9	104.3 ± 69.8	109.5 ± 73.4
LPA	29.9 ± 14.0	31.4 ± 15.4	30.5 ± 14.6
MVPA	5.7 ± 4.7	6.4 ± 5.3	6.0 ± 5.0
LPA + MVPA	35.6 ± 15.4	37.8 ± 17.2	36.6 ± 16.2
Prolonged sitting (> 30 min)	142.3 ± 90.5	161.8 ± 85.7	150.9 ± 88.9
Sit-stand transitions (n/sitting-hr)	7.8 ± 5.8	6.1 ± 6.6	7.0 ± 6.2
Total time (min per day)			
Sitting	619.8 ± 95.0	624.9 ± 87.4	622.1 ± 91.7
Standing	243.1 ± 82.9	236.4 ± 75.4	240.2 ± 79.7
LPA	79.5 ± 27.7	80.4 ± 28.3	79.9 ± 27.9
MVPA	17.7 ± 7.6	18.2 ± 7.2	17.9 ± 7.4
LPA + MVPA	97.1 ± 31.4	98.6 ± 31.9	97.8 ± 31.6
Prolonged sitting (> 30 min)	308.3 ± 107.2	322.5 ± 103.0	314.6 ± 105.5
Sit-stand transitions (n/sitting-hr)	6.0 ± 2.2	5.4 ± 1.9	5.7 ± 2.1
Cardiometabolic risk biomarkers			
CMR (sum of Z scores)	0.02 ± 0.7	−0.01 ± 0.6	0.00 ± 0.7
Fasting glucose (mg/dL)	96.5 ± 37.1	91.0 ± 14.1	94.1 ± 29.4
Fasting insulin (uU/mL)	77.6 ± 57.3	71.1 ± 55.0	74.7 ± 56.3
HDL-cholesterol (mg/dL)	60.1 ± 18.8	56.4 ± 16.5	58.5 ± 17.9
Triglycerides (mg/dL)	121.0 ± 74.4	120.3 ± 68.1	120.7 ± 71.7
Diastolic BP (mm Hg)	77.7 ± 10.7	77.4 ± 10.7	77.5 ± 10.7
Systolic BP (mm Hg)	124.9 ± 16.4	123.4 ± 15.7	124.3 ± 16.1
LDL-cholesterol (mg/dL)	110.8 ± 30.3	113.1 ± 34.1	111.8 ± 32.0
Weight (kg)	82.2 ± 22.8	82.9 ± 20.8	82.5 ± 21.9
BMI (kg/m2)	29.8 ± 7.6	28.7 ± 6.4	29.3 ± 7.1
Total body fat (%)	36.3 ± 9.6	32.8 ± 10.2	34.7 ± 10.0

*CMR* summary continuous metabolic risk score, *HDL* High-density lipoprotein, *BP* Blood pressure, *BMI* Body mass index

**Fig. 2:**
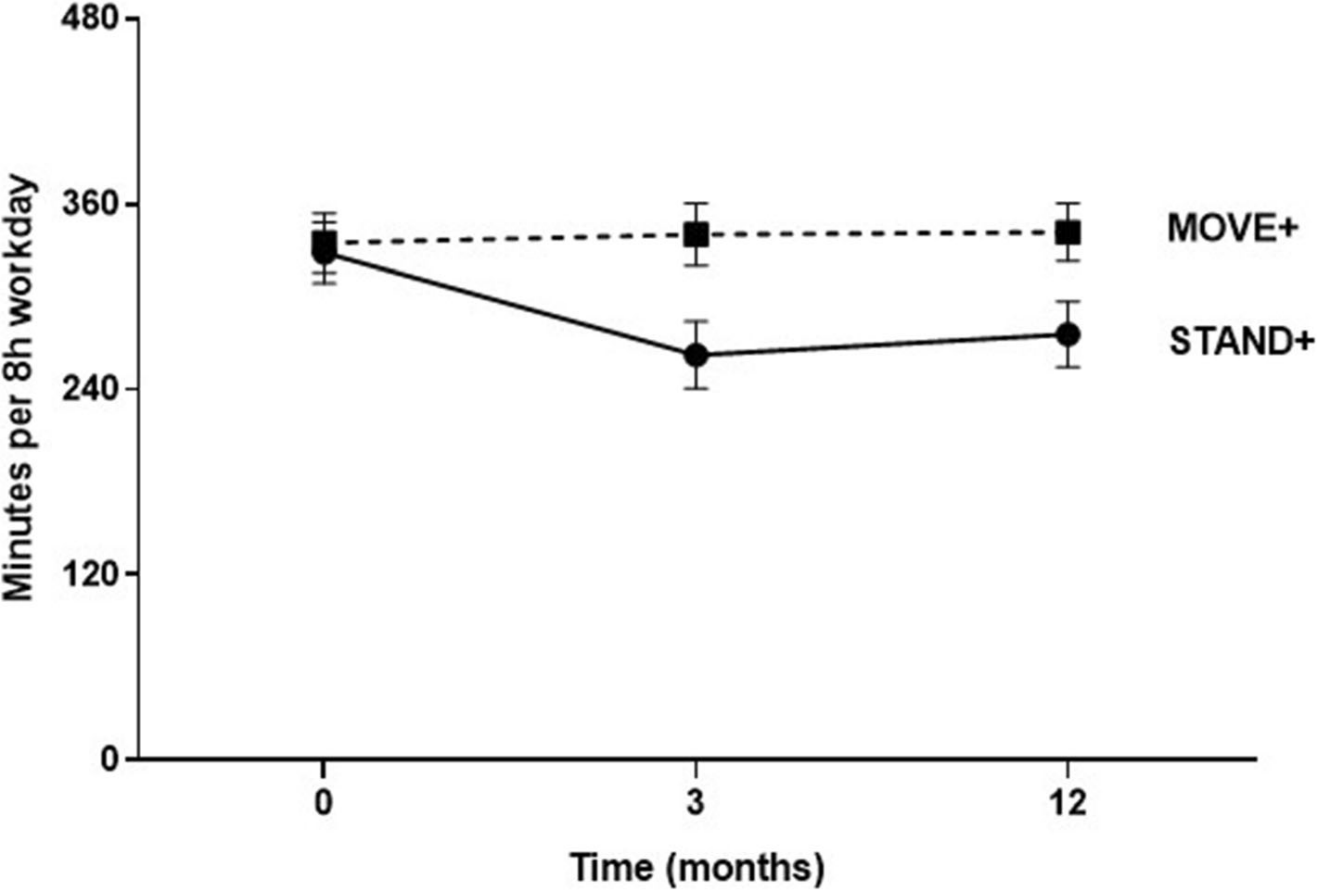
Workplace sitting time in STAND+ and MOVE+ study arms at 0, 3, and 12 months. Errors bars are 95% confidence intervals

Waking activPAL wear time was high: 92% of *MOVE+* participants had ≥5 valid days and 3 valid work periods (6.8 ± 1.1 valid days and 4.4 ± 1.0 work periods), with waking wear time of 15.3 ± 0.8 h per valid day and 8.3 + 0.8 h per valid work period. *STAND+* was similar in valid days (87%, 6.5 ± 1.3 valid days and 4.1 ± 1.0 work periods) and wear time (15.2 ± 0.9 h per valid day and 8.5 ± 1.0 h per valid work period). The results of the primary trial outcomes of activPAL-measured sitting and LPA, standardized to an 8 h work day, are shown in Table 2 at 3 and 12 months. The adjusted mean change in sitting time at 3 months for *STAND+* was − 63.8 ± 6.3 mins per 8 h workday, and for *MOVE+* was + 3.7 ± 6.6 mins per 8 h workday, giving a between arm difference of 67.5 ± 9.0 min per 8 h workday. This between-group difference was largely sustained at 12 months, 59.2 ± 7.4 min (Fig. 2). Nearly all the sitting reduction for *STAND+* was replaced by standing, as the 12-month difference in standing time was 49.4 ± 5.5 min per 8 h workday. Changes in LPA and MVPA activity at work were small and favored *STAND+*. Changes in total sitting time were modestly attenuated compared to work time only at 3 and 12 months. There were no appreciable changes in any activPAL-measured outcome when non-work time was analyzed separately (not shown).

**Table 2:** Intervention effects on objectively measured work time and total time activity variables, in minutes, at 3 and 12 months

	Time	*Stand+*	*Move+*	Difference (95% CI)	ICC
		Adjusted Mean Change (95% CI)	Adjusted Mean Change (95% CI)		
Primary Outcomes					
Work periods (*n* = 546)					
Sitting	3 M	−63.8 (−76.8,-50.7)	3.7 (−10.0, 17.4)	− 67.5 (−86.5,-48.4)	0.07
	12 M	−52.4 (− 62.9,-42.0)	6.8 (−4.3, 17.8)	−59.2 (− 74.6,-43.8)	0.03
LPA	3 M	−0.2 (− 1.6,1.2)	0.3 (− 1.2,1.9)	− 0.5 (−2.6, 1.6)	0.01
	12 M	2.0 (− 0.1,4.2)	− 0.2 (− 2.4,2.1)	2.2 (− 0.9,5.4)	0.06
Secondary Outcomes					
Work periods (*n* = 546)					
Standing	3 M	63.3 (50.7,75.8)	−4.0 (−17.1,9.1)	67.3 (48.9,85.6)	0.07
	12 M	49.4 (37.9,60.9)	−6.3 (−18.2,5.7)	55.7 (38.9,72.4)	0.05
MVPA	3 M	0.7 (0.1,1.3)	−0.2 (−0.8, 0.5)	0.9 (− 0.0, 1.8)	0.03
	12 M	1.0 (0.3, 1.6)	−0.4 (−1.1,0.3)	1.3 (0.4,2.3)	0.02
LPA + MVPA	3 M	0.5 (−1.3,2.4)	0.2 (− 1.8,2.2)	0.3 (−2.4,3.1)	0.03
	12 M	3.0 (0.7,5.4)	−0.5 (−3.0, 1.9)	3.6 (0.1,7.0)	0.05
Prolonged sitting (> 30 min)	3 M	− 32.3 (−44.9,-19.8)	7.9 (−5.2,21.0)	− 40.3 (− 58.6, − 22.0)	0.06
	12 M	− 26.8 (− 38.2, − 15.5)	19.0 (7.1, 30.8)	−45.8 (−62.4,-29.2)	0.04
Sit-stand transitions^a^	3 M	0.7 (0.1,1.2)	0.0 (− 0.6,0.6)	0.7 (− 0.1,1.5)	0.03
	12 M	0.8 (0.1,1.5)	−0.1 (− 0.7, 0.9)	0.7 (− 0.4,1.8)	0.01
Total time (*n* = 487)					
Sitting	3 M	−49.1 (−66.6,-31.6)	6.3 (−11.7,24.4)	−55.4 (−80.7,-30.2)	0.10
	12 M	−38.8 (−49.4,-28.2)	8.9 (−2.6,20.4)	− 47.7 (− 63.6,-31.7)	0.00
Standing	3 M	50.9 (35.5,66.3)	−4.5 (−20.4,11.4)	55.4 (33.1,77.6)	0.10
	12 M	39.0 (28.5,49.6)	−5.8 (−17.0,5.5)	44.8 (29.1, 60.5)	0.01
LPA	3 M	−2.0 (−5.9,1.9)	−1.5 (− 5.5,2.5)	−0.5 (−6.2,5.1)	0.09
	12 M	−0.4 (−4.6,3.6)	−3.0 (−7.3, 1.2)	2.6 (−3.4,8.5)	0.07
MVPA	3 M	0.2 (−0.7,1.1)	−0.4 (−1.4,0.5)	0.6 (− 0.7,2.0)	0.05
	12 M	0.5 (−0.2,1.2)	− 0.2 (− 0.9,0.6)	0.7 (− 0.4, 1.8)	0.00
LPA + MVPA	3 M	−1.8 (−6.4,2.8)	− 1.9 (− 6.7,2.9)	0.1 (− 6.6, 6.8)	0.10
	12 M	0.1 (−4.4,4.6)	−3.2 (− 7.9,1.5)	3.3 (− 3.3,9.8)	0.07
Prolonged sitting (> 30 min)	3 M	−26.8 (− 44.6,-9.0)	9.0 (−9.4,27.5)	−35.9 (− 61.7,-10.1)	0.09
	12 M	−22.2 (−34.7,-9.8)	19.2 (5.9,32.5)	−41.4 (− 60.0,-22.9)	0.01
Sit-stand transitions^a^	3 M	0.2 (− 0.1,0.5)	−0.2 (− 0.5,0.2)	0.4 (− 0.1,0.8)	0.11
	12 M	0.2 (−0.1,0.5)	− 0.3 (− 0.6,-0.0)	0.5 (− 0.1,0.9)	0.04

*3 M* 3 months, *12 M* 12 months, *LPA* Light-intensity physical activity, *MVPA* Moderate-vigorous physical activity. Work period outcomes have been standardized to an 8 h workday (minutes). ^a^Sit-stand transitions are expressed as number of transitions per sedentary hour

**Fig. 3:**
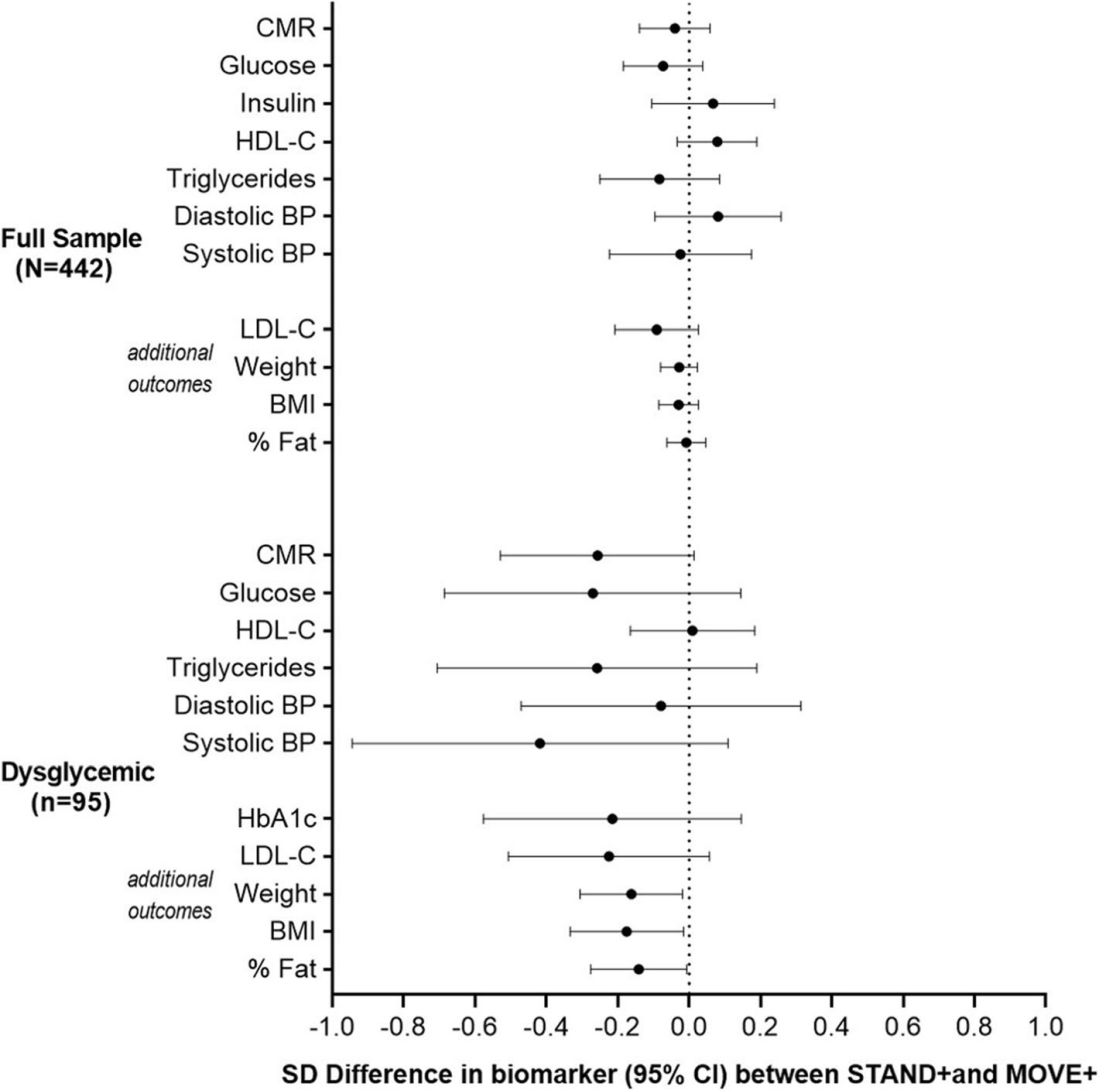
Between-arm differences in clustered metabolic risk score, its individual components, and additional risk biomarkers at 12 months in the full sample and exploratory dysglycemic subsample

The secondary outcome, clustered cardiometabolic risk score is shown in Fig. 3 in standardized effect sizes, along with the individual components of this score**.** Supplementary Table 3 includes the individual components of the risk score and other anthropometric and chronic disease risk factors in their original metrics. These effects, in the total sample between intervention arms, were small and not statistically significant but generally favored the *STAND+* arm. Restricting the analysis to the subset of dysglycemic individuals (*n* = 95), the effect sizes were larger for blood glucose, triglycerides, systolic blood pressure, glycated hemoglobin, LDL-cholesterol, body weight and body fat. The sitting and LPA changes were somewhat smaller in this subgroup relative to the full sample: adjusted between-arm mean change in sitting time at 12 months was − 42.8 ± 14.9 mins per 8 h workday, favoring *STAND+,* and LPA time at 12 months was + 1.8 ± 2.9 mins per 8 h workday, also favoring *STAND+*.

### Harms

No harms or adverse events were reported.

## Discussion

This trial provides strong evidence for the efficacy of sit-stand workstations over 12 months - when delivered alongside a multilevel intervention – for reducing sedentary time among office workers. Sitting at work was significantly decreased in *STAND+*, replaced by standing, compared to *MOVE+* using their routine sitting desk. The magnitude of these effects was large, ~ 1 h per workday. These effects materialized within 3 months and were maintained at 12 months. After accounting for non-work days, compensation with more sitting outside of work was negligible. Despite both groups engaged in active interventions to increase physical activity, effects were small and only present in *STAND+*. While there were no observed effects in the total sample on cardiometabolic risk, among the subgroup with prediabetes and diabetes (*n* = 95), we observed clinically meaningful changes for *STAND+* compared to *MOVE+* for blood glucose, glycated hemoglobin, triglycerides, LDL-cholesterol, systolic blood pressure, body weight, and body fat.

The results on cardiometabolic risk among dysglycemics require attention. The strength of effects were compelling and clinically meaningful, not unexpected given their higher risk. The effects may have significant public health implications for reducing cardiovascular disease risk and mortality [20]. These effects are biologically plausible, as there now exists a rich body of experimental studies indicating that replacing sitting time with standing and/or LPA augments insulin sensitivity and glucose disposal in the large lower body muscle groups for which frequent activation is required throughout the day when replacing sitting with standing and/or LPA [21–26]. Furthermore, postural changes that are required with relatively frequent use of sit-stand workstations may have beneficial peripheral vascular effects that could favorably impact cardiometabolic risk [27–29]. Large cohort studies have consistently found sedentary time in the general adult population to independently predict future risk of CVD and all-cause mortality [3, 30].

Our study appears to be the first to find an effect of reducing sedentary time at work with the use of sit-stand workstations on body weight. This effect was not trivial, with a between-arm effect of − 3.5 ± 1.5 kg favoring *STAND+* in the dysglycemic sample. Caution must be used in interpreting this finding because it was exploratory and limited to the dysglycemic subgroup. One potential explanation for this impact on body weight is that reducing sedentary time may require more energy expenditure for those with higher body weight. The intervention may also have been partially mediated by related behavior changes, including diet. Indeed, a prior pilot study of sit-stand workstation use found a decrease in energy intake during the active intervention period [31].

The current study is the largest and most diverse experimental study to date on the topic of reducing sedentary time in office workers with the use of sit-stand workstations. There are two other group randomized trials for comparison. In the *Stand Up Victoria* trial, Healy et al. [6] randomized 14 worksites (*N* workers = 231) to a 12-month intervention using sit-stand workstations or no-intervention control. They found similar reductions in sitting time over 12 months (− 44 min/8 h workday). Reductions in a cardiometabolic risk score were not significant, although fasting glucose reductions were significant (favoring worksites assigned to sit-stand workstations). In the *Stand More AT (SMArT) Work* trial, Edwardson et al. [10] randomized 37 worksites (*N* workers = 143) to a similar set of interventions and found − 41 min/8 h workday reductions in sitting time at 12 months. They also found improvements in health-related quality and improvements in work-related outcomes. Our results for sitting time reductions are consistent with these previous trials, where sitting time was primarily replaced with standing, with small but possibly systematic increases in physical activity during work. While sit-stand workstations afford desk-bound workers an option to continue their work while standing, increasing physical activity requires moving away from the desk and possibly disrupting work time. The current study bolsters the generalizability of the results of the previous studies because of a notably larger sample size and inclusion of a more diverse set of worksites. Both previous studies recruited worksites from single organizations, while *Stand and Move at Work* recruited 24 clusters from independent organizations across two states and three workplace sectors. Because of the larger sample size, meaningful sub-analyses of 95 dysglycemic individuals were possible.

The current trial did not include a non-intervention control group. Thus, we cannot compare our intervention results to similar worksites with no active intervention. However, this design enabled us to test whether sit-stand workstations are needed as part of a multilevel intervention that worksites could implement. The *MOVE+* intervention without the use of sit-stand workstations was ineffective relative to the *STAND+* intervention. This finding was consistent with the *Stand Up Victoria* and *SMArT at Work* interventions, both of which included non-intervention control arms. However, our study findings are not generalizable to all worksites. We only included full-time sedentary employees in relatively good health with no contraindications to standing and LPA. Strengths of our study included a large and diverse sample of adults across 24 worksites, two geographical regions, and three work sectors, as well as objective measurement of the primary and secondary outcomes. We also observed good 12-month adherence at the individual level. In fact, after accounting for routine workplace turnover the attrition rate was < 4%.

In summary, *Stand and Move at Work* is the largest and most comprehensive group-randomized trial to test the efficacy of sit-stand workstations to date. The results indicate that sit-stand workstations can reduce sitting time at work by approximately one hour/work day over 12 months. In those with prediabetes and diabetes, we observed trends towards clinical improvement in cardiometabolic risk and body weight. Future research should include more experimental studies including the population at high risk for diabetes and CVD.

## Supplementary information

**Additional file 1: Supplemental Table 1.** Baseline demographics of the analyzed sample by study arm.

**Additional file 2: Supplemental Table 2.** Baseline demographics of the dysglycemic sample by study arm.

**Additional file 3: Supplementary Table 3.** Intervention effects on cardiometabolic risk biomarkers at 12 months.

## Data Availability

The datasets supporting the conclusions of this article are available upon request though the corresponding author, Dr. Matthew Buman (ORCID ID 0000–0002–5130-3162).
