# Supplementary material for: Efficacy of the ‘Stand and Move at Work’ multicomponent workplace intervention to reduce sedentary time and improve cardiometabolic risk: a group randomized clinical trial
Source: Int J Behav Nutr Phys Act. 2020 Oct 27;17:133. doi: 10.1186/s12966-020-01033-3 (PMC7592578; doi:10.1186/s12966-020-01033-3)
Supplement: Supplementary file 1 — Additional file 1: Supplemental Table 1. Baseline demographics of the analyzed sample by study arm. [file 12966_2020_1033_MOESM1_ESM.pdf]

Supplemental Table 1. Baseline demographics of the analyzed sample by study arm.

	STAND+		*MOVE+*		Total	
	n	(%)	n	(%)	N	(%)
N worksites	12	(50.0)	12	(50.0)	24	(100.0)
N individuals	263	(54.0)	224	(46.0)	487	(100.0)
Region						
Phoenix, Arizona	137	(52.1)	102	(45.5)	239	(49.1)
Minneapolis/St. Paul, Minnesota	126	(47.9)	122	(54.5)	248	(50.9)
Age (years)	46.6	±10.9	43.7	±10.6	45.3	±10.8
Race						
Non-Hispanic White	183	(69.6)	169	(75.5)	352	(72.3)
Hispanic	37	(14.1)	26	(11.6)	63	(12.9)
Non-Hispanic Black	15	(5.7)	6	(2.7)	21	(4.3)
Non-Hispanic Asian	12	(4.6)	14	(6.3)	26	(5.3)
Other/Multiracial/Unknown	16	(6.1)	9	(4.0)	25	(5.1)
Female	220	(83.7)	134	(59.8)	354	(72.7)
Education						
Less than college	10	(3.8)	13	(5.8)	23	(4.7)
College/Some college	178	(67.7)	127	(56.7)	305	(62.6)
Graduate/Professional	68	(25.9)	78	(34.8)	146	(30.0)
Unknown	7	(2.7)	6	(2.7)	13	(2.7)
Work sector						
Academic	95	(36.1)	76	(33.9)	171	(35.1)
Industry/healthcare	82	(31.2)	66	(29.5)	148	(30.4)
Government	86	(32.7)	82	(36.6)	168	(34.5)
Job type						
Executive	35	(13.3)	31	(13.8)	66	(13.6)
Professional	141	(53.6)	133	(59.4)	274	(56.3)
Clerical	83	(31.6)	56	(25.0)	139	(28.5)
Behavioral Outcomes						
Work periods (min per 8 h workday)						
Sitting	328.6	±80.9	334.8	±73.7	331.4	±77.7
Standing	115.2	±76.5	107.3	±71.6	111.6	±74.3
LPA	30.6	±14.9	31.3	±15.6	31.0	±15.2
MVPA	5.6	±4.7	6.5	±5.5	6.0	±5.1
LPA + MVPA	36.3	±16.3	37.9	±17.5	37.0	±16.9
Prolonged sitting (>30 min)	141.2	±90.5	156.7	±85.4	148.3	±88.4
Sit-stand transitions (n/sitting-hr)	7.9	±5.9	6.1	±6.7	7.1	±6.3
Total time (min per day)						
Sitting	615.9	±97.7	617.9	±87.7	616.8	±93.2
Standing	245.2	±85.4	242.0	±76.7	243.8	±81.4
LPA	81.4	±28.6	81.5	±28.8	81.5	±28.6
MVPA	17.5	±7.8	18.6	±7.3	18.0	±7.6
LPA + MVPA	98.9	±32.4	100.1	±32.6	99.4	±32.4
Prolonged sitting (>30 min)	306.4	±109.3	314.4	±99.1	310.1	±104.7
Sit-stand transitions (n/sitting-hr)	6.1	±2.2	5.4	±1.9	5.7	±2.1
Cardiometabolic risk biomarkers						
Total sample						
CMR (sum of Z scores)	0.00	±0.7	0.01	±0.6	0.01	±0.7
Fasting glucose (mg/dL)	95.3	±33.8	91.5	±14.2	93.5	±26.6
Fasting insulin (uU/mL)	77.6	±59.1	70.7	±50.8	74.4	±55.5
HDL-cholesterol (mg/dL)	60.3	±17.9	56.3	±16.8	58.4	±17.5
Triglycerides (mg/dL)	117.5	±64.8	123.7	±71.0	120.4	±67.7
Diastolic BP (mm Hg)	77.8	±10.7	77.7	±10.7	77.8	±10.7
Systolic BP (mm Hg)	125.4	±15.9	124.1	±15.9	124.8	±15.9
LDL-cholesterol (mg/dL)	110.7	±29.4	114.5	±34.4	112.5	±31.8
Weight (kg)	82.6	±22.5	83.5	±20.7	83	±21.6
BMI (kg/m2)	29.9	±7.6	28.8	±6.4	29.4	±7.1
Total body fat (%)	36.7	±9.6	32.6	±10.3	34.8	±10.2

CMR = summary continuous metabolic risk score; HDL = high-density lipoprotein; BP = blood pressure; BMI = body mass index.
