# Supplementary material for: Efficacy of the ‘Stand and Move at Work’ multicomponent workplace intervention to reduce sedentary time and improve cardiometabolic risk: a group randomized clinical trial
Source: Int J Behav Nutr Phys Act. 2020 Oct 27;17:133. doi: 10.1186/s12966-020-01033-3 (PMC7592578; doi:10.1186/s12966-020-01033-3)
Supplement: Supplementary file 2 — Additional file 2: Supplemental Table 2. Baseline demographics of the dysglycemic sample by study arm. [file 12966_2020_1033_MOESM2_ESM.pdf]

Supplemental Table 2. Baseline demographics of the dysglycemic sample by study arm.

	STAND+		*MOVE+*		Total	
	n	(%)	n	(%)	N	(%)
Dysglycemic subgroup						
n individuals	57	(60.0)	38	(40.0)	95	(100.0)
CMR (sum of Z scores)	0.7	±0.8	0.5	±0.7	0.6	±0.8
Fasting glucose (mg/dL)	129.5	±66.8	111.8	±20.0	122.4	±53.8
HDL-cholesterol (mg/dL)	52.4	±14.1	52.7	±18.7	52.5	±16.0
Triglycerides (mg/dL)	142.4	±70.7	159.9	±98.5	149.4	±82.9
Diastolic BP (mm Hg)	83.4	±10.4	81.4	±11.2	82.6	±10.7
Systolic BP (mm Hg)	135.5	±15.8	131.7	±18.7	134.0	±17.0
HbA1c	6.2	±1.4	5.7	±0.7	6.0	±1.2
LDL-cholesterol (mg/dL)	118.0	±30.6	116.5	±34.2	117.4	±31.9
Weight (kg)	98.0	±24.0	94.1	±22.1	96.5	±23.2
BMI (kg/m2)	35.3	±8.4	32.1	±6.9	34.0	±8.0
Total body fat (%)	42.0	±9.3	36.9	±10.2	40.0	±9.9

CMR = summary continuous metabolic risk score; HDL = high-density lipoprotein; BP = blood pressure; BMI = body mass index.
