# Supplementary material for: Efficacy of the ‘Stand and Move at Work’ multicomponent workplace intervention to reduce sedentary time and improve cardiometabolic risk: a group randomized clinical trial
Source: Int J Behav Nutr Phys Act. 2020 Oct 27;17:133. doi: 10.1186/s12966-020-01033-3 (PMC7592578; doi:10.1186/s12966-020-01033-3)
Supplement: Supplementary file 3 — Additional file 3: Supplementary Table 3. Intervention effects on cardiometabolic risk biomarkers at 12 months. [file 12966_2020_1033_MOESM3_ESM.pdf]

Supplementary Table 3. Intervention effects on cardiometabolic risk biomarkers at 12 months

	STAND+	*MOVE+*		
	Adjusted Mean Change (95% CI)	Adjusted Mean Change (95% CI)	Difference, *STAND+* - *MOVE+* (95% CI)	ICC
Total (n=444)				
CMR (sum of Z scores)	-0.05 (-0.09,-0.00)	-0.02 (-0.07,0.03)	-0.03 (-0.10,0.04)	0.01
Fasting glucose (mg/dL)	-0.7 (-2.7,1.3)	1.3 (-0.8, 3.4)	-2.0 (-4.9, 1.0)	0.02
Fasting insulin (uU/mL)	-2.7 (-9.2,3.8)	-6.4 (-13.3,0.4)	3.7 (-5.8,13.3)	0.04
HDL-cholesterol (mg/dL)	1.0 (-0.3,2.3)	-0.3 (-1.8,1.1)	1.4 (-0.6,3.3)	0.01
Triglycerides (mg/dL)	-2.9 (-10.6,4.9)	2.8 (-5.4,10.9)	-5.6 (-17.0,5.7)	0.03
Diastolic BP (mm Hg)	0.1 (-1.2,1.4)	-0.7 (-2.1,0.6)	0.9 (-1.0,2.8)	0.06
Systolic BP (mm Hg)	-2.1 (-4.3,0.1)	-1.7 (-4.0,0.5)	-0.4 (-3.5,2.8)	0.08
Additional outcomes				
LDL-cholesterol (mg/dL)	0.5 (-2.0,2.9)	3.3 (0.7,6.0)	-2.9 (-6.6, 0.8)	0.00
Dysglycemic subgroup (n=95)				
CMR (sum of Z scores)	-0.15 (-0.27,-0.04)	0.02 (-0.12, 0.17)	-0.18 (-0.37, 0.01)	0.03
Fasting glucose (mg/dL)	-6.6 (-13.4,0.3)	0.6 (-7.6, 8.9)	-7.2 (-18.2,3.9)	0.02
HDL-cholesterol (mg/dL)	0.3 (-1.6, 2.2)	0.1 (-2.2, 2.4)	0.2 (-2.9,3.2)	0.00
Triglycerides (mg/dL)	-13.4 (-33.5,6.7)	4.0 (-18.2,26.2)	-17.5 (-47.8,12.9)	0.23
Diastolic BP (mm Hg)	-0.6 (-3.4,2.2)	0.2 (-2.8,3.3)	-0.8 (-5.0, 3.4)	0.21
Systolic BP (mm Hg)	-5.1 (-10.8,0.6)	1.5 (-4.5,7.5)	-6.6 (-15.0,1.7)	0.29
Additional outcomes				
HbA1c (%)	-0.0 (-0.3,0.2)	0.2 (-0.1,0.5)	-0.3 (-0.7,0.2)	0.22
LDL-cholesterol (mg/dL)	-3.6 (-9.1,1.8)	3.5 (-3.3,10.3)	-7.1 (-16.1,1.8)	0.00
Weight (kg)	-1.9 (-3.9,0.1)	1.6 (-0.7,3.9)	-3.5 (-6.6,-0.4)	0.03
BMI (kg/m2)	-0.7 (-1.4,0.0)	0.5 (-0.3,1.4)	-1.2 (-2.4,-0.1)	0.01
Total body fat (%)	-0.9 (-1.8,-0.0)	0.5 (-0.5,1.5)	-1.4 (-2.8,-0.1)	0.00

CMR = summary continuous metabolic risk score; HDL = high-density lipoprotein; BP = blood pressure; BMI = body mass index.
